# Bridging the gap: a translational perspective in spinal cord injury

**DOI:** 10.3389/ebm.2024.10266

**Published:** 2024-09-26

**Authors:** Omar Imad Hassan, Soichiro Takamiya, Azam Asgarihafshejani, Michael G. Fehlings

**Affiliations:** ^1^ Division of Genetics and Development, Krembil Brain Institute, University Health Network, Toronto, ON, Canada; ^2^ Institute of Medical Science, University of Toronto, Toronto, ON, Canada; ^3^ Division of Neurosurgery, Krembil Neuroscience Centre, Toronto Western Hospital, University Health Network, Toronto, ON, Canada; ^4^ Division of Neurosurgery and Spine Program, Department of Surgery, University of Toronto, Toronto, ON, Canada

**Keywords:** spinal cord injury, neuroregeneration, animal models, clinical trials, pathophysiology

## Abstract

Traumatic spinal cord injury (SCI) is a devastating and complex condition to treat with no curative options. In the past few decades, rapid advancements in our understanding of SCI pathophysiology as well as the mergence of new treatments has created more optimism. Focusing on clinical translation, this paper provides a comprehensive overview of SCI through its epidemiology, pathophysiology, currently employed management strategies, and emerging therapeutic approaches. Additionally, it emphasizes the importance of addressing the heavy quality of life (QoL) challenges faced by SCI patients and their desires, providing a basis to tailor patient-centric forms of care. Furthermore, this paper discusses the frequently encountered barriers in translation from preclinical models to clinical settings. It also seeks to summarize significant completed and ongoing SCI clinical trials focused on neuroprotective and neuroregenerative strategies. While developing a cohesive regenerative treatment strategy remains challenging, even modest improvements in sensory and motor function can offer meaningful benefits and motivation for patients coping with this highly debilitating condition.

## Impact statement

Despite advancements in medical, surgical, and rehabilitation management for traumatic spinal cord injury (SCI), there remains a critical need for neuroprotective and neuromodulatory treatment strategies. By providing an overview of the current state of SCI understanding and management strategies, this paper aims to bridge the gap between current therapeutic limitations and emergent treatments. It also examines the challenges in treating and studying SCI due to the complexities in the heterogeneity of the disease. Emphasizing the integration of patient feedback and emergent therapies, this paper advocates for the development of tailored approaches that are crucial for advancing SCI care and inclusivity. Ultimately, the goal is to provide insights and guidance that will enhance recovery and quality of life outcomes for SCI patients, benefiting researchers, healthcare professionals, policymakers, and caregivers alike.

## Introduction

Traumatic spinal cord injury (SCI) remains a debilitating condition, but over the past century rapid growth has been made to uncover its pathophysiology and translate preclinical research to patient care. This paper provides an overview of SCI pathophysiology, epidemiology, currently employed management strategies, and emerging therapeutic approaches. It also highlights the quality of life (QoL) challenges faced by patients as well as their desires, providing a basis for caretakers to tailor more patient-centric forms of care. This review underscores the heterogeneous nature of SCI both in disease presentation and individual patient needs, having profound effects on treatment effectiveness. By delving into the wide range of strategies to manage SCI, both established in the clinic and emerging approaches, this paper examines their therapeutic potential and limitations. Furthermore, this paper discusses the frequently encountered barriers in translation from preclinical models to clinical settings. Although the need remains urgent for novel and effective SCI treatments, there is great hope with the continued progress in the field aimed at enhancing QoL and functional outcomes for patients.

## Epidemiology

Of those that survive the initial injury, most will have persisting neurological deficits [1]. Direct costs incurred by SCI due to permanent disability are large, estimated to be between 1.1 and 4.6 million USD per patient in the United States [2]. The World Health Organization estimates that 250,000 to 500,000 people suffer a new SCI each year [3] but direct comparisons are shrouded by a lack of an international standard for SCI reporting. Despite challenges surrounding SCI reporting, commonalities can still be drawn from regionally reported data. Within developed countries, SCIs are primarily caused by motor vehicle accidents (MVAs), yet there is a shift towards an increase in fall-related injuries [1, 4, 5]. For example, in the United States, 38.1% of injuries were caused by MVAs from 2010 to 2014, with falls as the close second cause at 31.0% [6].

In regard to sex, males make up the highest distribution of SCI at 79.8% as opposed to female SCI cases at 20.2% [6]. Within the elderly population, this disparity between sex decreases as the age at which SCI occurs in females tends to be later [7, 8]. Preclinical models for SCI assessing the role of gonadal hormones do not have an established consensus [7]. Clinically, the higher incidence of male SCI as well as disparities in the cause and types of injuries make sex-based comparisons difficult.

With an aging global population, the average age of injury is increasing from patients in their late 20s to those in their early 40s [2]. This increase in age remains true for most causes of SCI, with the exception of violence, as it predominantly occurs in younger individuals (16–30 years of age) [6]. In comparison to younger patients, individuals over 50 have greater rates of cervical injury leading to paraplegia than their younger counterparts [9].

There are also variations in injury trends between countries, predominantly related to economic status. Developing countries primarily report falls as the leading cause of SCI, while MVAs dominate SCI cases in wealthier nations [5, 10–12]. However, there are exceptions to this trend. Prevention efforts have reduced MVAs, work-related SCIs, and driving-related injuries in high-income countries. Unfortunately, MVAs and work-related SCIs remain significant issues in low and middle-income countries. Advances in acute surgical, medical, and rehabilitation care have disproportionately benefited high-income countries [5, 10–12].

Despite its stronger economic position in the world, falls are beginning to dominate the SCI landscape in Japan due to the large elderly population [13]. Violence also contributes to a greater proportion of SCI cases in developing regions [14]. Even within a nation, variations in urbanization, economic status, and occupation have different outcomes [15].

Mortality and quality of life in patients with SCI have improved but remain lower than in healthy, age-matched controls in the global population [16]. In the first-year post-injury, the mortality rate is close to 3.8%, followed by 1.2% the next year and an increased rate of 1.2% per annum over the next 10 years [16]. The most significant indicators of mortality in the time surrounding the injury are the severity of the SCI as well as the level of the SCI and the age of the patient [16–18]. Major risks that consistently place patients at higher long-term mortality rates are a loss of autonomy as well as reduced social engagement and support [19, 20].

## Pathophysiology

**FIGURE 1:**
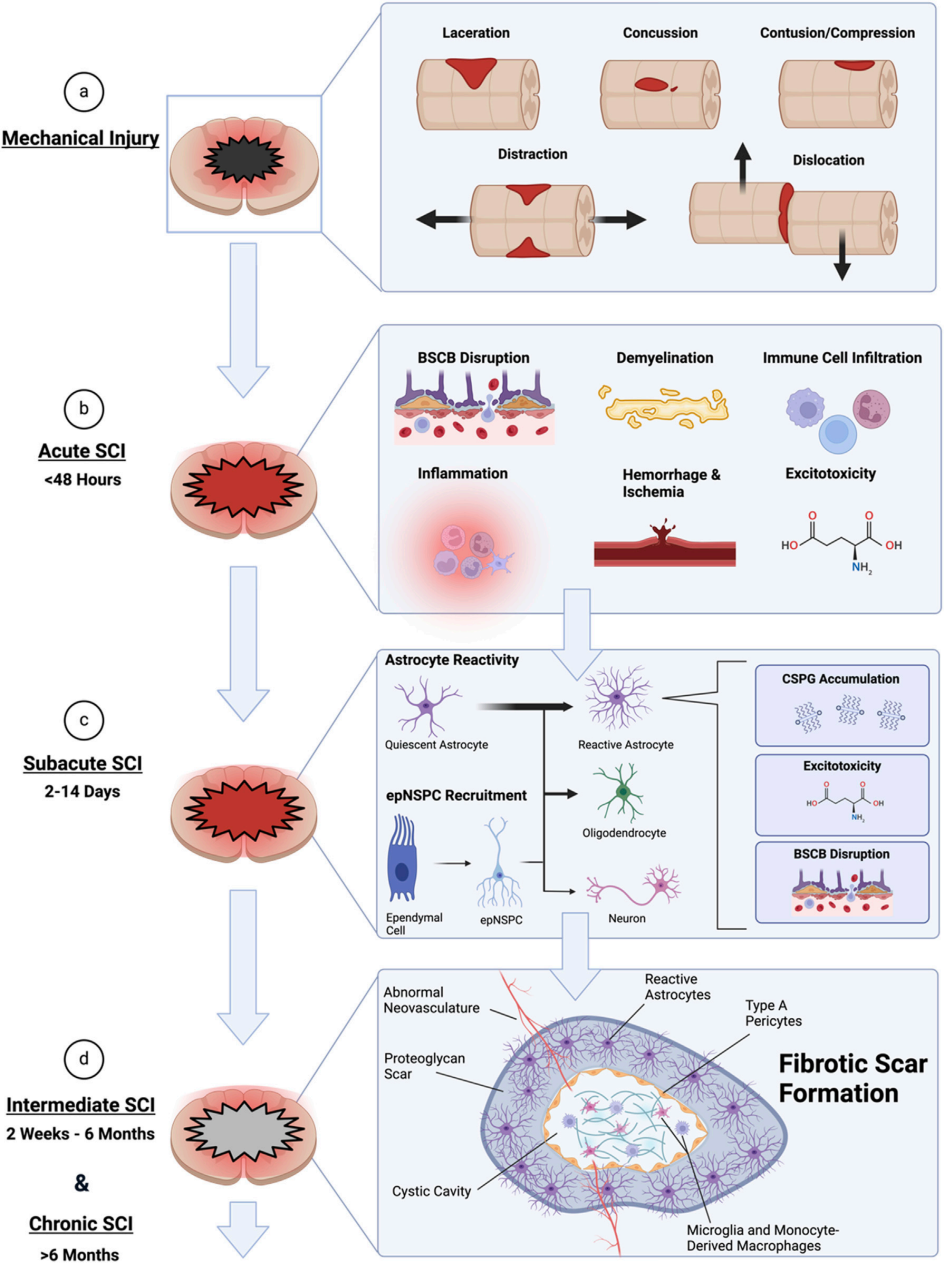
Timeline of the pathophysiological developments of spinal cord injury (SCI). **(A)** The initial mechanical forces that contribute to lesion formation and necrosis, initiating SCI. **(B)** The acute phase of injury occurs following the initial injury. It is characterized by inflammation, ischemia, blood-spinal cord barrier (BSCB) disruption, immune cell infiltration and recruitment, demyelination, as well as excitotoxicity. This leads to further damage of the parenchyma beyond the initial lesion. **(C)** The subacute phase sees the recruitment of astrocytes from their quiescent state to reactive. Astrocytes are also derived from resident ependymal cells through neural stem/progenitor cell (epNSPC) differentiation. Predominantly epNSPCs differentiate into astrocytes, with few becoming oligodendrocytes and even less becoming neurons. The reactive astrocytes then contribute to further disruption of the BSCB, reduced glutamate uptake involved in excitotoxicity, and chondroitin sulfate proteoglycan (CSPG) deposition. Inflammation and ischemia also persist in this phase. **(D)** During intermediate and chronic phases of SCI, the reactive astrocyte border and fibrotic scar is formed and consolidated. The fibrotic scar contains type A pericytes, abnormal vasculature growth, and CSPG deposits. The scarring and cystic formation inhibits recovery. Created with Biorender.com.

SCI is a heterogeneous and multifaceted condition that threatens the physical, social, and vocational well-being of patients. It is one of the leading causes of paralysis worldwide [1, 21]. SCI begins with an external mechanical trauma that causes contusion and compression of the spinal cord (Figure 1). This leads to the generation of toxic debris and disruption of vasculature, which initiates the secondary injury cascade [22]. In the acute phase (<48 h post-injury), inflammation is initiated accompanying the activation of microglia into a proinflammatory phenotype, which leads to glutamate excitotoxicity and nitric oxide production [1, 23, 24]. Furthermore, blood-spinal cord barrier (BSCB) disruption, hemorrhage, ischemia, as well as demyelination contribute to greater neuronal and glial cell death [25, 26]. The subsequent subacute phase (2–14 days post-injury) sees sustained inflammation and ischemia as well as the recruitment of resident astrocytes into reactive astrocytes [27, 28]. These astrocytes have impaired glutamate reuptake contributing to excitotoxicity, disrupted BSCB contact and maintenance, as well as formed chondroitin sulfate proteoglycan (CSPGs) deposits that disrupt regeneration [25]. Ependymal cells undergo significant alterations after SCI. This involves activating specific signaling pathways in the spinal cord that promote self-renewal, proliferation, and differentiation. An orchestrated regulation of receptor and ion channel expression fine-tunes and coordinates the activation of ependymal cells after SCI or cell transplantation [29]. While ependymal cells have been proposed as adult neural stem cells, controversy remains as to whether they provide a significant portion of scar-forming astrocytes to protect tissue and function after SCI [30]. Infiltrating microglia is also a key cellular component in orchestrating the glial scar that develops after SCI to protect neural tissue [31].

Resident ependymal cells are recruited during this period and form neural stem/progenitor cells (epNSPCs) that predominantly differentiate into astrocytes [32, 33], contributing to the upregulated population of reactive astrocytes. In the intermediate (2 weeks-6 months post-injury) and chronic phases of SCI (>6 months post-injury), a fibrotic scar core consisting of type A pericytes, abnormal neovasculature, and CSPGs is formed within a reactive astrocyte encasing border [34]. Scarring, cystic cavity formation, as well as limited remyelination and axon regrowth act in concert to greatly stunt recovery. This causes devastating and often permanent neurological deficits with complex barriers to treatment.

## Targets for potential SCI treatment

### BSCB disruption

The BSCB is a special structure within the spinal cord parenchyma that mediates the exchange of compounds between the blood and the parenchyma while maintaining a regulated chemical balance and homeostasis crucial for neural function [35–37]. Preserving the integrity of the BSCB may enhance spinal cord repair and functional improvement, therefore, the BSCB plays a role in the pathophysiology of SCI progression [34–36].

Morphological and functional changes in the BSCB after SCI include vascular changes, increased permeability of the BSCB, edema, and cavity formation [38]. The initial mechanical damage, combined with compression, laceration, and distraction, contributes to disruption of the neurovascular system [35]. The adverse environment then rapidly results in neuropil damage, swollen neurovascular unit cells, and membrane structure disruption [32, 36, 37]. The morphological alterations accompanying BSCB disruption are instrumental in the progression of SCI because the disrupted BSCB allows the immune cells to enter the injured sites [35, 39, 40].

Lymphocytic infiltration mediates inflammation, reactive astrogliosis, scar formation, and neutrophils leading to demyelinating as well as neuroinflammatory events [39, 40]. The BSCB alterations following SCI lead to altered permeability, which commences several minutes after injury, persists for up to 4 weeks, and may extend over a longer duration, often accompanied by cavity formation [41, 42]. In addition, following SCI, edema begins within several minutes, intensifies rapidly, and persists for up to 15 days, affecting both the lesion site and adjacent segments [38, 39]. Progressive cavity formation causes deficits in neurological function and neuropathic pain [43–45].

### Inflammation

Although inflammation serves as a vital defense mechanism in removing pathogens, clearing debris, and facilitating wound healing in the context of SCI, it also accentuates detrimental effects [45, 46] Following SCI, the inflammatory response leads to the production of toxic molecules which, instead of aiding in healing, cause further damage on otherwise intact tissues. While inflammation is required for repair, the response that follows SCI is often exaggerated and leads to further damage and cell loss [47]. Further adding to the complexity of SCI, the infiltration of immune cells non-resident to the CNS (central nervous system) also plays a critical role in inflammation and signaling molecules, affecting the progression of the disease. These infiltrating immune cells are guided by cytokines produced from astrocytes, microglia, peripherally derived macrophages, and endothelial cells [48, 49].

Following SCI, Microglia change their cellular morphology and protein expression profiles [49–51]. Under normal physiological conditions, microglia have long, thin processes that extend from the central cell body to sample the extracellular environment [50, 51]. After SCI, microglia retract their processes and assume an amoeboid shape, primed for phagocytosis and debris clearance [49–51]. In the first hours after injury, microglia, astrocytes, and neurons synthesize pro-inflammatory cytokines [49, 52]. Chemokines drive the increased expression of selectins and cell adhesion proteins on endothelial cells, facilitating integrin-mediated adhesion of circulating immune cells and the subsequent leakage of monocytes and neutrophils into the spinal cord [53, 54]. As the injury response progresses, microglia proliferate extensively during the first 2 weeks, accumulating around the lesion site. These activated microglia position themselves at the interface between infiltrating leukocytes and astrocytes, orchestrating glial scar formation by releasing factors such as IGF-1 [31].

Infiltrating macrophages provide proteolytic enzymes, reactive oxygen species, and inflammatory cytokines to the injury microenvironment but also perform the necessary functions of debris clearance, cellular remodeling, and producing pro-regenerative factors [55, 56]. Preclinical studies have shown that while macrophages increase axon regeneration and neuronal function, they can also worsen tissue destruction. The dual beneficial and reparative functions of macrophages make understanding their role in the injury response difficult [55, 56].

### Ischemia and hemorrhage

Mechanical damage from SCI leads to the disruption of capillaries and the BSCB, which creates a harsh microenvironment for spinal cord parenchyma [57]. A direct rupture of the local capillaries induces bleeding into the parenchyma of the spinal cord, which could cause increased release of cytokines and chemokines from macrophages, microglia, and astrocytes into the extracellular space [57]. The presence of red blood cells in the parenchyma is likely to induce free radicals and consequently lead to edema [58, 59]. On the contrary, neural tissue edema can also increase interstitial pressure, which presses the neighboring vessels and causes ischemia [60]. The lack of adenosine triphosphate caused by ischemia and ion channel defects results in an ion imbalance [60, 61].

### Demyelination and re-myelination

Oligodendrocytes oversee the generation and maintenance of myelin segments, which is crucial to maintaining the integrity of axons and eases axon signal conduction [62, 63]. After SCI, mechanical damage and the imbalance of local microenvironment factors leads to demyelination [64, 65]. The apoptosis of oligodendrocytes is potentially the leading cause of axonal demyelination [64, 65]. The level of oligodendrocyte apoptosis at the epicenter of the lesion peaks within a week of the injury; however, uninjured axons around the lesion remain myelinated [64, 65]. The presence of myelin debris inhibits remyelination, thus the extent and quality of remyelination are limited [66].

Mechanical injury, ischemia, inflammatory cytokines, oxidative stress, excitotoxicity, and autophagy can cause the death of oligodendrocytes because of demyelination and remyelination imbalance [67–69]. Molecules involved in demyelination are potential inhibitors of axon regeneration, thus the process of demyelination inhibits the regeneration of axons [67–69].

Following a SCI, remyelination mainly involves replacing oligodendrocytes, with the primary source of these new cells being progenitor oligodendrocytes and endogenous neural stem cells [67]. Endogenous neural stem cells remain inactive in normal conditions and become activated upon spinal cord damage; these cells mainly differentiate into astrocytes and to a lesser degree into oligodendrocytes [67, 70]. This suppression of differentiation into oligodendrocytes is mainly due to the lack of growth factors that switch the balance toward differentiation into oligodendrocytes [67, 70].

### Hyperexcitation (switch from KCC2 to NKCC1)

NKCC1 and KCC2 are members of the SLC12 cation-chloride co-transporter (CCC) family, which participate in physiological and pathophysiological processes by regulating intracellular and extracellular chloride concentrations, and in turn the GABAergic system [71, 72]. NKCC1 transports Cl^−^ into cells while KCC2 transports Cl^−^ out of cells, thereby regulating chloride balance and neuronal excitability. An imbalance of NKCC1 and KCC2 after SCI will disrupt CI^−^ homeostasis, resulting in the transformation of GABA neurons from an inhibitory to an excitatory state, which leads to abnormal conditions such as spasticity and neuropathic pain [73–75].

After SCI, the segment below the injury site presents a state similar to upregulation of NKCC1 seen in the early stages of development [75]; therefore, the expression of KCC2 was reported to be downregulated at the injury site, followed by a transient upregulation of NKCC1 expression levels, and this altered expression trend was consistent with the post-neuropathic pain occurrence [76].

Inflammation or injury can inhibit the expression and function of KCC2 in the dorsal horn and advance the development of neuropathic pain [77, 78]. GABAA receptors (GABAARs) are involved in the regulation of tonic inhibition in the dorsal horn, sustaining the relative balance of inhibition and excitation in the central nervous system [79]. After SCI, the function of GABAARs changes and their activation can cause a depolarizing shift as well as the disclosure of nociceptive sensitization [80]. Therefore, improving the abnormal Cl− concentration gradient in the dorsal horn through targeting KCC2 and NKCC1 represents a promising therapeutic direction for restoring the inhibitory function of the GABAergic system and relieving or improving neuropathic pain [81–83]. Moreover, disruption of Cl− homeostasis after SCI, especially the downregulation of KCC2 in motor neurons, depolarizes the Cl− equilibrium potential and decreases the strength of postsynaptic inhibition [74].

Numerous studies have confirmed the therapeutic effect of NKCC1 and KCC2 in neuropathic pain, spasticity, and motor functional recovery post SCI, and these co-transporters are expected to become key targets in future SCI treatments [74, 84]. However, KCC2 and NKCC1 are distributed throughout the nervous system and methods to achieve localization, orientation, and quantitative regulation of their levels may be the main obstacle to their clinical application in the treatment of SCI.

## Patient-centric approaches

The concept of patient-centered care (PCC) is crucial for bridging the gap between patients’ desires and what healthcare professionals consider beneficial for patients. Gerteis et al. identified that patients defined PCC as having the following dimensions: 1) respect for patient’s values, preferences and expressed needs; 2) coordination of care and integration of services within an institutional setting; 3) communication between the patient and providers; 4) dissemination of accurate, timely and appropriate information; 5) education about the long-term implications of disease and illness; 6) physical care, comfort and the alleviation of pain; 7) emotional support and alleviation of fears and anxiety; 8) involvement of family and friends; and 9) transition and continuity from one locus of care to another [85]. This section will delve into specific dimensions of PCC relevant to patients with SCI, with a focus on translational perspectives.

### Respect for patients’ desires

Healthcare professionals generally strive to provide the best treatment for patients with SCI in accordance with clinical practice guidelines [86]. However, therapeutic strategies for SCI do not always align with patient satisfaction. According to a qualitative study on decision-making regarding bladder drainage methods after SCI, conducted by Engkasan et al., some patients felt that they were forced to accept their doctor’s decision [87]. Additionally, Scheel-Sailer et al. reported in their qualitative interview-based study that patients with SCI often experience difficulties making decisions during the initial rehabilitation phase due to physical, psychological, and environmental factors [88]. Thus, it appears that patients’ opportunities for decision-making in therapeutic strategies for SCI might be limited in certain contexts.

Importantly, patients’ treatment preferences might differ from the actual treatments, potentially undermining respect for their desires. Bowers et al. conducted a survey to clarify SCI patients’ preferences regarding methylprednisolone sodium succinate (MPSS) treatment for acute SCI and found that most SCI patients considered MPSS treatment important, even if it offered only minor neurological benefits and carried a risk of complications [89]. However, the 24-hour administration of high-dose MPSS to adult patients within 8 h of SCI is still controversial, with only a weak recommendation in the 2017 AO Spine Clinical Practice Guidelines [86]; therefore, not all patients wishing to receive this treatment may be able to, depending on the physicians’ decision.

Looking forward, as novel therapeutic strategies for SCI emerge, they will initially lack robust evidence to guide evidence-based decision-making. In such situations, it will be important for physicians to respect patient’s desires and provide them with opportunities to make decisions about their treatments.

### Accessibility of information

Patients with SCI have a keen interest in health-related information; hence, the accessibility of such information is crucial for them [90]. According to the interest assessment survey performed by Edwards et al. in the early 2000s, 64% of Canadian chronic SCI patients reported using the Internet to obtain research information [91]. In a more recent 2020 study by Farrehi et al., 89% of participants with SCI in the United States reported sourcing information about experimental therapies online [92]. This trend suggests that access to medical information, including emerging therapies, will continue to grow in the future. However, SCI patients tend to deem information from SCI specialists as more reliable [92]. Therefore, it is equally important to enhance accessibility to SCI specialists, and there are opportunities to leverage emerging areas, such as Telemedicine, to improve access for patients in rural areas [93].

Moreover, improving accessibility for research information also benefits researchers by aiding in the recruitment of participants for clinical trials, as individuals with SCI are willing to participate in translational research [94].

### Enhancing quality of life for patients

Among the various neurological symptoms experienced after SCI, pain is the most prevalent issue of SCI as highlighted by patient feedback, followed by bowel and bladder dysfunction, spasticity, and sexual dysfunction [95, 96]. It significantly affects patients’ quality of life by interfering with sleep and daily activities [95]. A survey by Jensen et al. found that pain was both the most common (experienced by 84% of individuals) and the most severe symptom among participants [96]. According to a meta-analysis regarding the prevalence of neuropathic pain following SCI, the pooled point prevalence rate was 53% [97]. However, a postal survey by Finnerup et al. indicated that only a small number of patients received treatment with antidepressants or anticonvulsants, which are considered to be most effective for neuropathic pain [98]. This suggests that there is significant potential for enhancing the quality of life in SCI patients.

In preclinical studies, behavioral assessment tests for sensory function are less commonly utilized than those for motor function. A systematic review regarding animal models of SCI showed that sensory tests, such as the von Frey filament test, were used in only 16.3% of studies, compared to 89.2% for locomotor tests [99]. This discrepancy might be due to most researchers focusing primarily on motor function. However, considering the clinical application and the impact on patients’ quality of life, there may be merit in the inclusion of sensory assessment as well as locomotor assessment.

## Currently employed strategies in the management of SCI

### Early surgical decompression

Surgical decompression of the spinal cord within the first 24 h after injury limits tissue damage by restoring compromised blood flow and reducing the extent of ischemia-related secondary injuries. A recent pooled analysis to evaluate the efficacy of early decompressive surgery for SCI demonstrated that the American Spinal Injury Association (ASIA) motor score in the early (within 24 h of SCI) surgery group was significantly higher than that in the late (after 24 h of SCI) surgery group (23.7 points vs. 19.7 points; *p* = 0.0006) at 1 year after injury [100]. According to the latest meta-analysis regarding timing of decompressive surgery for acute SCI, patients were 2 times more likely to recover by ≥2 grades on the ASIA Impairment Score at 6 months and 1 year after SCI (risk ratios: 2.76 [95% CI: 1.60–4.98] and 1.95 [95% CI: 1.26–3.18]) if they underwent decompressive surgery within 24 h after injury [101]. Based on this evidence, the recommendation for early surgical decompression (within 24 h after SCI) was upgraded in the recently published AO Spine-Praxis Clinical Practice Guidelines from “Quality of Evidence: Low; Strength of Recommendation: Weak” in 2017 [86] to “Quality of Evidence: Moderate; Strength of Recommendation: Strong” in 2024 [101]. Although the evidence has become stronger, early surgical decompression for acute SCI remains a significant challenge in low- and middle-income countries due to limited logistical and infrastructural resources [102].

As for ultra-early surgical interventions (within 4, 5, 8, and 12 h after SCI), it is difficult to draw firm conclusions on their efficacies compared to early surgical decompression, due to the inconsistency in results observed so far [101]. Just as the evidence for early surgical decompression has been established, the evidence for ultra-early surgical intervention is expected to be solidified as more clinical findings become available.

Recently, a phase III RCT, Duroplasty for Injured Cervical Spinal Cord with Uncontrolled Swelling (DISCUS) (NCT04936620), was initiated. This ongoing trial compares laminectomy with duroplasty to laminectomy alone for treating acute cervical SCI. It is expected to reveal the optimal surgical procedure for acute SCI in the near future.

### Blood pressure augmentation

Hemodynamic management following acute SCI is crucial, as ischemia and hypoperfusion can exacerbate secondary injury. Pre-clinical research has indicated that maintaining arterial pressure can improve spinal cord blood flow and, consequently, electrophysiological function [103, 104]. Accordingly, the 2013 guidelines from the American Association of Neurosurgical Surgeons (AANS) and the Congress of Neurological Surgeons recommended maintaining a mean arterial pressure (MAP) of 85–90 mmHg for the first 7 days post-SCI [105]. However, considering the strict MAP target range of 5 mmHg and newer literatures since the 2013 AANS/Congress of Neurological Surgeons guidelines, the 2024 AO Spine Guideline now recommends that MAP should be maintained between 75 and 80 mmHg as a lower limit and not exceed 90–95 mmHg at the higher range during the first 3–7 days post-SCI [106]. A phase III, randomized, controlled trial (RCT), the Randomized Trial of Early Hemodynamic Management of Patients Following Acute Spinal Cord Injury (TEMPLE) (NCT02232165), was initiated in 2017. This ongoing trial aims to compare augmented blood pressure management (targeting MAP of 85–90 mmHg) with conventional management (65–70 mmHg), and may provide additional evidence on the benefit of blood pressure augmentation for acute SCI.

Additionally, spinal cord perfusion pressure (SCPP), which has recently emerged as a more relevant parameter to predict functional outcomes as compared to MAP, is recommended to be maintained above 50 mmHg [107]. It is anticipated that the ongoing Canadian-American Spinal Cord Perfusion Pressure and Biomarker Study (CASPER) (NCT03911492) will soon provide further evidence to support this approach.

### Methylprednisolone sodium succinate (MPSS)

MPSS is a corticosteroid that inhibits lipid peroxidation of the neuronal membrane and prevents secondary damage of SCI [108]. The National Acute SCI Study (NASCIS) trials were representative trials of MPSS for SCI. In the NASCIS-2 trial, the primary analysis did not show significant motor recovery in the MPSS group; however, secondary analyses demonstrated that patients who had received high-dose MPSS within 8 h post-SCI improved motor scores compared to the control group at 6 months post-SCI (16.0 points vs. 11.2 points; *p* = 0.033) [109]. Additionally, the NASCIS-3 trial suggested that patients who received MPSS within 3 h post-SCI should be maintained on the 24-hour treatment regimen, whereas those who received 3–8 h after SCI should be maintained on the 48-hour therapy [110]. Although there are some controversies from a perspective of complications [111], the side effects of steroids are much less relevant in modern times with improved general medical care and the avoidance of steroids in medically compromised individuals. Currently, a 24-hour infusion of high-dose MPSS should be offered to adult patients with acute SCI (<8 h post-injury) as a treatment option [86].

## Challenges in translation

Generally, the process from technology initiation to FDA approval in translational science takes a considerable amount of time. McNamee et al. reported that the median interval from technology initiation to establishment was 25 years, to the start of clinical trials was 29 years, and to the first FDA approval was 36 years among new molecular entities approved by FDA between 2010 and 2014 [112]. Broadly, clinical and translational research encompasses the following five phases: T0, basic research (pre-clinical research); T1, translating basic research to humans (phase I clinical trials); T2, translating findings to patients (phase II/III clinical trials); T3, translating research to general practice care (phase IV clinical trials); and T4, translating research to populations or communities [113]. This section will focus on animal models for phase T0, highlight key clinical trials for phase T1–2, and examples of advanced translation.

### Animal models and clinical relevance

In translational research, multiple animal models should be used to verify the effectiveness of potential treatments and establish proof of concept. Utilizing a variety of models enhances the robustness and translatability of the research findings [114]. When considering differences among preclinical SCI models, it is important to note the animal species, injury mechanisms, and injured level.

#### Animal species

Rodent models are the predominant model in SCI research. Rats are most commonly used (72.4%) in SCI preclinical research, followed by mice at 16% [99]. Rodent models also have distinct phases of SCI pathophysiology that are clinically relevant and, given their rapid reproduction cycle and small size, can allow for greater sample sizes. This is especially useful in drug studies where multiple groups are needed to test a range of dosages for safety and efficacy. The preference for rat models is due to their long-term usage as a robust and reliable model for assessing even incremental improvements [115]. Immunodeficient rats that lack T-cell presence have allowed for cell transplantation therapy experiments without the risk of host-vs-graft disease. As an example, human pluripotent stem cell lines have been applied in these models with success [116]. The main caveat to this model, however, is that immunosuppression of SCI patients to allow for transplantation would be through immunosuppressant drugs rather than genetic alterations. This hinders translatability but also reduces variability from the many possible tailored immunosuppressant regimes. For more extensive genetically modified animal research, mouse models are utilized due to their widespread usage in knockout studies. Immunodeficient mice with engrafted human hematopoietic stem cells have shown promise as a translatable model for human immune responses after SCI [117]. However, they are less resilient to SCI induction with higher mortality rates and have species-specific timelines in SCI less reflective of human patient timelines. Where rats have T-cell infiltration peak at 3–7 days post-injury, closer to the human timeline of 7–9 days post injury [49], mice do not have significant T-cell infiltration until after 14 days post injury [49].

Despite the advantages of rodent models, there is a need for large animal and non-human primate models from a translational perspective [118, 119]. When comparing SCI in rodents and humans, functional recovery after injury in rodent models tends to be much faster compared to humans, which seems to be associated with various neural pathways. For example, the rubrospinal tract has been reported as an alternative pathway to improve motor function after corticospinal tract injury in rodent models, which is not observed in humans [120, 121]. Moreover, the size of the spinal cord and its surrounding environment, including the cerebrospinal fluid, differ considerably in rodents, potentially affecting the distribution of locally delivered therapies [122]. This holds especially true for the development of surgical interventions, which are limited when applied to the small stature of rodent models. By examining both rodent and large preclinical animal models, more robust findings can be obtained prior to clinical trials. To date, various large animal SCI models have been utilized, including those involving pigs [123], dogs [124], cats [125], and monkeys [126].

#### Injury mechanisms

Based on the mechanisms of injury, SCI models can be classified as contusion, transection, compression, and distraction/dislocation models. Contusion models are the most commonly used (43.4%), followed by transection (34.4%) and compression models (20.5%) [99].

Contusion models are created using weight-drop apparatuses or electromagnetic impactors, such as the New York University impactor [127] and the infinite horizon impactor [128]. Compression models are generated by compression using a modified aneurysmal clip [129], forceps [130], or balloon [131]. Both contusion and compression models effectively reflect the pathophysiology of SCI; in particular, clip contusion models not only cause compression but also contusion and hypoperfusion, closely mimicking clinical situations [118]. Transection models, which include complete and partial transection, are advantageous for investigating axonal regeneration following SCI. However, they do not fully represent the complex pathophysiology of SCI, as the spinal cord is seldom sharply transected in clinical settings [118].

#### Injured levels

Although approximately 60% of SCIs occur at the cervical level [132], only 12% of preclinical research studies have utilized cervical models, with the majority (over 80%) employing thoracic SCI models [99]. This discrepancy may stem from the challenge of postoperative care in cervical SCI models, including manual bladder expression and feeding as well as daily fluid administration, which is necessary to maintain low mortality rates [133]. While challenging to implement, it is essential to validate therapeutic effects in cervical models, as the pathophysiology of cervical SCI differs from that of thoracic SCI due to anatomical and physiological variations [134].

Even though the animal models can be sophisticated from a clinical relevance perspective, a large gap between preclinical studies and early-phase clinical trials remains due to issues of poor validity and reproducibility, often caused by improper study designs. The lack of alignment in design between basic research and clinical trials, including the dosage of drugs and timing of administration, makes it difficult to predict the effectiveness of novel therapies in human trials [135]. To maximize clinical translation, we should refine study designs as well as using clinically relevant animal models.

## Regenerative strategies under research

### Stem cells and associated growth factors

#### Endogenous stem cells

Cellular replacement strategies are necessary to restore disrupted neural signaling pathways following the extensive parenchyma loss after SCI. Oligodendrocyte progenitor cells (OPCs) are the majority of progenitor cells that proliferate and differentiate in response to SCI [136]. OPCs are unipotent and neural stem/progenitor cells (NSPCs) are fewer, underlying the spinal cord’s limited neurogenic potential [137]. Of the NSPCs, the largest population responding to SCI are ependymal derived neural/stem progenitor cells (epNSPCs) in the spinal canal. They display multipotent properties and are capable of self-renewal, responding to SCI in the acute phase through proliferation and migration to the site of injury. Although they have been shown *in vitro* to have the capability to differentiate into neurons, astrocytes, and oligodendrocytes, *in vivo* studies have displayed them to be particularly biased towards an astrocytic fate post-SCI, with a small potential for becoming oligodendrocytes and an even lower potential to form neurons [137, 138].

Strategies to bias epNSPCs towards neuronal cell fates are emerging. Bioartificial scaffoldings to bias NSPCs towards neuronal cells such as those developed by Zhang et al. [139] have been shown to facilitate neural stem cells to differentiate into neuron-like cells. As an alternative approach, biasing through changes to the microenvironment has also been displayed. Ohori et al. [140] injected fibroblast growth factor 2 and epidermal growth factor within the lesion site in a rat SCI model to promote immature markers of neuronal cells in NSPCs. As a caveat, they used NSPCs that were genetically manipulated by a retrovirus, pMXIG, to express Neurogenin2 (NGN2) and Mash1, transcription factors that bias towards neurons and oligodendrocytes.

#### Induced pluripotent stem cells

Induced pluripotent stem cells (IPSCs) can be biased as neural progenitor cells (NPCs) for transplantation and integration into the spared parenchyma to enhance local circuits and aid in motor recovery [141]. These cells can be made in an autologous fashion from fibroblasts, circumventing the need for immunosuppressants when using exogenous cells as well as the ethical concerns of their origins. Fibroblasts are exposed to the factors Oct4, Sox2, Klf2, and c-Myc in accordance with the work done by Yamanaka and Takahashi on mice in 2006 [142] and in human fibroblasts by Takahashi et al. in 2007 [143]. This comes at the cost of time, however, for the development and biasing of IPSCs, limiting their application to later stages of injury.

Generating IPSCs also comes with added risks, particularly related to tumorigenicity. When generating the IPSCs, residual undifferentiated cells can proliferate and form tumors [144], thus emphasizing the need for stringent quality control.

One of the factors used to generate IPSCs from fibroblasts, c-Myc, is protooncogenic and often overexpressed in a majority of human cancers, contributing to over 40% of tumor formations [145, 146]. Retroviral c-Myc introduction has also shown an increased tumorigenicity in mouse models [147]. Clinically this carries a large risk. Using alternative factors reduces the efficiency and speed of IPSC induction but makes the IPSCs clinically acceptable [148].

The first IPSCs generated by Yamanaka and Takahashi through retrovirus transduction resulted in random integration [142, 143] at start sequences with increased likelihoods of loss of function effects [149]. Alternative strategies have arisen with lowered tumorigenicity. Sendai and adeno-associated viruses, as well as plasmid integration, have been used to potentially lower teratogenicity but are highly inefficient [150–152]. Sustained delivery of synthetic mRNA that encodes for the reprogramming factors used by Yamanka’s group are more efficient and avoid the heritable tumorigenicity of cellular DNA modification [153, 154]. Direct reprogramming of adult somatic cells without viral vectors through the transient expression of Msi1, Ngn2, and MBD2 by Ahlfors et al. has shown high reprogramming efficiency, no tumorgenicity in murine models, and low-cost [155]. It remains hopeful for clinical application.

## Overview of current significant clinical trials

In the currently employed therapies for SCI, there are not enough neuroprotective and neuro regenerative approaches. To address this issue, numerous clinical trials have been performed. In this section, we provide an overview of clinical trials examining neuroprotective (Table 1), regenerative (Table 2), and other strategies for SCI.

**TABLE 1:** Summary of leading completed and ongoing clinical trials regarding neuroprotective therapies for spinal cord injury.

Drug	Treatment Type	Study design	Study period	Stage of SCI	Sample size	Outcome	References
Minocycline	Anti-apoptosis	Phase II, RCT	2004–2008	Acute (<12 h of SCI)	52	A trend toward motor improvement in cervical SCI patients treated with minocycline was observed No significant difference in motor function in thoracic SCI patients treated with minocycline was observed	Casha et al. [156]
Riluzole	Anti-excitotoxicity	Phase IIb/III, RCT	2014–2021	Acute (<12 h of SCI)	193	All subgroups of cervical SCI patients treated with riluzole showed significant gains in functional recovery on the *post hoc* analyses	Fehlings et al. [157]
G-CSF	Anti-apoptosis, anti-inflammation	Phase III, RCT	2015–2019	Acute (<48 h of SCI)	88	A trend toward motor improvement in the G-CSF group was observed	Koda et al. [158]
KP-100 (HGF)	Anti-apoptosis, cell growth	Phase I/II, RCT	2014–2018	Acute (2–5 days post-SCI)	43	KP-100 contributed to motor improvement	Nagoshi et al. [159]
		Phase III, open-label, single-arm	2020–2023	Acute (<72 h of SCI)	25	not published yet	NCT04475224

Abbreviations: SCI, spinal cord injury; RCT, randomized controlled trial; G-CSF, granulocyte colony-stimulating factor; HGF, hepatocyte growth factor.

**TABLE 2:** Summary of leading completed and ongoing clinical trials regarding regenerative therapies for spinal cord injury.

Cell-based approach								
Cell type		Treatment type	Study design	Study period	Stage of SCI	Sample size	Outcome	References
NP/PCs		Anti-apoptosis, anti-inflammation (acute phase) Remyelination, axonal regrowth (chronic)	Phase I/IIa, open-label, single-arm	2021– (ongoing)	Subacute (7–60 days post-SCI)	5 (estimated)	Not completed yet	NCT04812431
			Phase I, open-label, single-arm	2020– (ongoing)	Subacute (14–28 days post-SCI)	4 (estimated)	Not completed yet	jRCTa031190228
OPCs		Anti-apoptosis, anti-inflammation (acute phase) remyelination, axonal regrowth (chronic)	Phase I/IIa, open-label, single-arm	2015–2018	Subacute (21–42 days post-SCI)	25	Two grade 3 serious adverse events (CSF leakage and bacterial infection) were observed 24/25 participants experienced functional recovery	Fessler et al. [168]
MSCs	Bone marrow-derived	Anti-apoptosis, anti-inflammation (acute phase) remyelination, axonal regrowth (chronic)	Phase II, open-label, single-arm	2014–2017	Subacute (26–54 days post-SCI)	13	No serious adverse events were observed 12/13 participants experienced functional recovery	Honmou et al. [169]
			Phase II/III, RCT, delayed-start	2022– (ongoing)	subacute (6–10 weeks post-SCI)	16 (estimated)	Not completed yet	NCT03935724
	Umbilical cord-derived		Phase I/II, RCT	2022– (ongoing)	Acute (<7 days post-SCI)	80 (estimated)	Not completed yet	NCT05693181
	Adipose tissue-derived		Phase I, open-label, single-arm	2017–2021	chronic (2–12 months post-SCI)	10	No serious adverse events were observed 7/10 participants experienced functional recovery	Bydon et al. [170]
	Muse cells		Phase II, open-label, single-arm	2019–2023	Subacute (<2 weeks post-SCI)	10	Not published yet	jRCT1080224764

Non-cell-based approach							
Drug	Treatment Type	Study Design	Study Period	Stage of SCI	Sample Size	Outcome	References
C3 transferase	Axonal growth, regeneration	Phase IIb/III, RCT	2016–2018	Acute (<72 h of SCI)	67	No significant difference in motor function in the cethrin group was observed	Fehlings et al. [171]
Anti-Nogo-A antibody	Axonal growth, regeneration	Phase II, RCT	2019–2023	Acute (4–28 days post-SCI)	129	Not published yet	NCT03935321
Endothelin B receptor agonist	Anti-apoptosis, enhances neuronal differentiation	Phase II, RCT	2019– (ongoing)	Acute (<48 h of SCI)	40 (estimated)	Not completed yet	NCT04054414
Anti-RGMa antibody	Axonal growth, regeneration	Phase II, RCT	2020– (ongoing)	Acute (<24 h of SCI)	54 (estimated)	Not completed yet	NCT04295538
		Phase II, RCT	2021– (ongoing)	Acute	72 (estimated)	Not completed yet	NCT04683848

Abbreviations: SCI, spinal cord injury; RCT, randomized controlled trial; NP/PCs, neural stem/progenitor cells; OPCs, oligodendrocyte progenitor cells; CSF, cerebrospinal fluid; MSCs, mesenchymal stem cells; RGMa, repulsive guidance molecule A.

### Neuroprotective therapy

#### Minocycline

Minocycline is a tetracycline antibiotic used clinically as an antimicrobial agent. It also exhibits anti-apoptotic characteristics through the inhibition of caspase-1 and –3 [160, 161]. A phase II RCT (NCT00559494) conducted from 2004 to 2008 demonstrated safety and a trend toward motor function improvement, as measured by the ASIA motor score, in cervical SCI patients treated with minocycline (14 points; 95% CI: 0–28; *p* = 0.05) [156]. Based on these promising results, a phase III RCT, Minocycline in Acute Spinal Cord Injury (MACS) (NCT01828203), was initiated in 2013. However, this trial was discontinued, and its results have not yet been published.

#### Granulocyte colony stimulating factor (G-CSF)

G-CSF, known as a growth factor for hematopoietic cells, has also demonstrated neuroprotective characteristics for SCI through angiogenesis, inflammation suppression, and apoptosis inhibition in preclinical research [162–164]. An open-label phase I/IIa trial of G-CSF for acute SCI conducted from 2008 to 2010 revealed no severe adverse events related to G-CSF administration [165]. Another open-label, non-randomized controlled phase II trial carried out between 2009 and 2011 by the same group found a significantly greater improvement in the ASIA motor score in the G-CSF group compared to the control group [166]. Encouraged by these promising results, a phase III RCT, the G-CSF mediated spinal cord injury recovery induction trial (G-SPIRIT) (UMIN000018752), was initiated in 2015. Although this trial reported no significant differences in the primary efficacy endpoint, measured by changes in the ASIA motor score at 3 months post-intervention between the G-CSF and control groups, those at 6 and 12 months showed a trend towards better improvement in the G-CSF group [158].

#### Hepatocyte growth factor

Hepatocyte growth factor (HGF) is secreted by mesenchymal stem cells and regulates cell growth and cell motility by activating a tyrosine kinase signaling cascade through binding to the c-Met receptor. In preclinical research using primate models, HGF enhanced motor neuron survival and reduced cavitation at the injured site [167]. A phase I/II RCT using recombinant human HGF (KP-100IT) for acute SCI (NCT02193334) was conducted starting in 2014. It demonstrated safety and suggested improvements in motor function as evaluated by the ASIA motor score [159]. Based on these results, an open-label, single-arm phase III trial (NCT04475224) was conducted. However, it appears not to have achieved its primary efficacy endpoints, potentially influenced by variations in patients’ baselines due to the COVID-19 pandemic. Detailed results, including any *post hoc* analyses, are to be published in the near future.

### Regenerative therapy (Table 2)

#### Cell-based therapy

Cell-based therapies are a promising strategy for the treatment of SCI, offering a variety of therapeutic mechanisms. Supported by substantial pre-clinical evidence, numerous clinical trials have been conducted.

##### Neural stem/progenitor cells (NS/PCs)

NS/PCs, capable of self-renewal and differentiating into neurons and glial cells, have been utilized in several clinical trials. Two clinical trials include an open-label phase I/II trial for chronic thoracic SCI (NCT01321333) and a single-blinded phase II RCT for chronic cervical SCI (NCT02163876), where human fetal brain-derived NS/PCs were transplanted into the spinal cord around the epicenter. These trials indicated no serious adverse events related to the intramedullary injection or additional spinal cord damage; however, they failed to demonstrate the efficacy anticipated by the sponsor [172–174]. Another open-label, single-arm phase I trial with the intramedullary transplantation of human spinal cord-derived NS/PCs for chronic thoracic SCI (NCT01772810) began in 2014, with 5-year follow-up results recently reported. According to this report, no serious adverse events were directly attributed to cell transplantation [175]. Currently, there are ongoing clinical trials using human embryonic stem cell (ESC)-derived NS/PCs and human induced pluripotent stem cell (iPSC)-derived NS/PCs. An open-label, single-arm phase I/IIa trial with human ESC-derived NS/PCs (NCT04812431) is targeting subacute cervical SCI and is estimated to be completed by 2028. Another open-label, single-arm phase I trial using human iPSC-derived NS/PCs (UMIN000035074, jRCTa031190228) is targeting subacute cervical or thoracic SCI [176]. This trial is expected to be completed by 2024 and aims to address ethical concerns associated with deriving NS/PCs from human ESC or fetuses.

##### Oligodendrocyte progenitor cells (OPCs)

OPCs are also self-renewing, multipotent cells that preferentially differentiate into oligodendrocytes, as opposed to NS/PCs. Preclinical studies have demonstrated their capability to secrete neurotrophic factors, suppress inflammation, remyelinate axons, and spare tissues [177–180]. An open-label, single-arm phase I trial (NCT01217008) was conducted from 2010 to 2013, involving the direct transplantation of OPCs into the injured epicenter in patients with subacute thoracic SCI. This study confirmed their safety for up to 10 years post-SCI [181]. Based on this safety profile, an open-label, single-arm phase I/IIa trial for subacute cervical SCI (NCT02302157) took place from 2015 to 2018. Results from this trial indicated not only the safety of OPCs but also functional improvements as assessed by the International Standards for Neurological Classification of Spinal Cord Injury examination at 1-year post-SCI [168]. Consequently, a phase III trial to confirm their efficacy is now warranted.

##### Schwann cells (SCs)

SCs have shown the ability to promote remyelination, improve axonal sparing, and reduce the inflammatory response in preclinical studies [182–184]. Two open-label, single-arm phase I trials of SCs for SCI have been conducted by the Miami Project to Cure Paralysis. The first one (NCT01739023) was performed between 2012 and 2015 and enrolled six patients with subacute thoracic SCI [185]. Another trial (NCT02354625) was conducted from 2015 to 2019 and enrolled six patients with chronic (more than 1 year) thoracic SCI [186]. In both trials, SCs were harvested from the sural nerve of the participants, and autologously transplanted into the epicenter of SCI, with no serious adverse events being reported. However, no evidence of its efficacy has been reported to date.

##### Mesenchymal stem cells (MSCs)

MSCs can exert immunomodulatory, anti-inflammatory, neuroprotective, and angiogenic effects by secreting numerous trophic factors [187], This secretion improves the local environment of the injured spinal cord. Due to their ability to migrate to the injured lesion [188], MSCs can be transplanted directly into the injured site or via intravenous injection, offering a less invasive option for patients.

MSCs can be derived from multiple sources, including bone marrow (BM), umbilical cord (UC), adipose tissue (AD), Wharton’s jelly, and amnion. Their efficacy has been demonstrated in several preclinical studies [189–193]. Based on these sources, numerous clinical trials have been conducted. For instance, autologous BM-MSCs were intravenously injected in an open-label, single-arm phase II trial for subacute cervical SCI (JMA-IIA00154). This trial reported no serious adverse events related to the cell injection and showed neurological improvement [169]. Based on the results, the MSC product (Stemirac^®^) has been approved through the conditional early approval program in Japan, although further evaluations are required to determine its efficacy. Additionally, a double-blinded, placebo-controlled, and delayed-start phase II/III trial, Stem Cells in Spinal Cord Injury (SCI2) (NCT03935724), began in 2022. In this trial, patients with subacute cervical and thoracic SCI are intrathecally injected with the BM-MSC product (Neuro-Cells). The trial is expected to be completed by 2024.

Regarding UC-MSCs, allogeneic UC-MSCs were administered intravenously in an open-label phase I/IIa RCT for acute SCI (NCT04331405). This trial showed only two mild adverse events (transitory mild hyperthermia after cell infusion) and most patients experienced neurological improvement, although the results from the phase IIa trial are still pending [194]. Currently, the same group is conducting a single-blinded phase I/II RCT for acute SCI, Systemic Umbilical Cord Blood Administration in Patients with Acute Severe Contusion Spinal Cord Injury II (SUBSCI II) (NCT05693181), which began in 2022 and is estimated to be completed by 2025.

As for AD-MSCs, an open-label, single-arm phase I trial, the Adipose Stem Cells for Traumatic Spinal Cord Injury (CELLTOP) (NCT03308565), was conducted at the Mayo Clinic from 2017 to 2021. In this trial, participants with subacute and chronic SCI intrathecally received AD-MSCs. Recent results indicated no serious adverse events, with 7 out of 10 participants showing improvement in AIS grade post-injection [170]. Another phase I/II RCT for acute thoracic SCI (NCT02917291) involved transplanting allogenic AD-MSCs (FAB117-HC) into the injured spinal cord. This trial was expected to be completed by 2023, though its current status is unknown.

Multilineage-differentiating stress-enduring (Muse) cells, identified as stress-tolerant pluripotent stem cells within MSCs, are promising for SCI therapy [195]. Muse cells recognize injured sites with sphingosine-1-phosphate (S1P) via the S1P receptor 2 and migrate accordingly. A preclinical study showed that they reduced cystic cavities and preserved axons [196]. An open-label single-arm phase II trial using Muse cells (CL2020) for acute/subacute cervical SCI (jRCT1080224764) was conducted in Japan. The trial was completed in 2023, and the results are expected to be reported in the near future.

#### C3 transferase

C3 transferase inhibits Rho signaling, consequently promoting axonal growth and regeneration [197, 198]. An open-label, single-arm, phase I/IIa trial (NCT00500812) was conducted from 2005 to 2009, and Cethrin, a recombinant C3 transferase, was applied to the surface of the dura mater overlying the injured lesion during decompressive surgery [199]. No serious adverse events were reported, and then, a phase IIb/III RCT, SPinal Cord Injury Rho INhibition InvestiGation (SPRING), was conducted from 2016 to 2018 (NCT02669849). Unfortunately, this trial was terminated because the interim efficacy results, evaluated by the upper-extremity motor score, did not show a significant difference between the C3 transferase group and the placebo [171].

#### Anti-Nogo-A antibody

Nogo-A is one of the myelin-associated proteins that inhibit neuronal growth by activating the Rho/ROCK pathway upon binding to the Nogo receptor [200]. Therefore, the anti-Nogo-A antibody has the potential to improve axonal regrowth by mediating Rho/ROCK signaling, as demonstrated in preclinical research using primate models [201]. An open-label phase I trial utilizing recombinant anti-Nogo-A antibody (ATI355) for acute traumatic SCI (NCT00406016) was performed from 2006 to 2011 and found no drug-related serious adverse events [202]. A phase II RCT trial using anti-Nogo-A antibody (NG-101) for acute cervical SCI, Nogo Inhibition in Spinal Cord Injury (NISCI) (NCT03935321), was initiated in 2019 and recently completed in 2023. In this trial, the anti-Nogo-A antibody treatment did not show a statistically significant benefit for the primary efficacy endpoint. The detailed results, including *post hoc* analysis, have not yet been published. Similarly, a phase I/II trial using a soluble Nogo-Receptor-Fc decoy (AXER-204) for chronic cervical SCI, ReNetX Safety Efficacy and Tolerability of AXER-204 for Chronic SCI (RESET) (NCT03989440), was conducted from 2019 to 2022. It demonstrated safety; however, no significant differences were observed for secondary efficacy endpoints between the AXER-204 group and the placebo group [203].

#### Endothelin B receptor agonist

Sovateltide, also known as IRL-1620 or PMZ-1620, is an endothelin B receptor agonist that has enhanced neuronal differentiation and reduced apoptosis in animal models of cerebral infarction [204, 205]. Following the promising results of a RCT with Sovateltide for acute cerebral ischemic stroke [206], a phase II RCT of PMZ-1620 for acute SCI (NCT04054414) was initiated in 2019. The trial is currently ongoing and is estimated to be completed in 2024.

#### Anti-repulsive guidance molecule A (RGMa) antibody

RGMa is a protein that activates the RhoA-Rho kinase pathway and consequently inhibits axonal regeneration [207]. In preclinical research using a primate model of SCI, an RGMa antibody facilitated the recovery of manual dexterity by enhancing the penetration of corticospinal tract fibers into laminae VII and IX [208]. To date, a few clinical trials are ongoing. Phase II RCT using a human anti-RGMa monoclonal antibody, known as Elezanumab (ABT-555), for acute SCI (NCT04295538) was initiated in 2020 and is estimated to be completed in 2026. Additionally, the expanded access program for Elezanumab has been approved (NCT04278235). Another phase II RCT using MT-3921 for acute cervical SCI (NCT04683848) was started in 2021 and is estimated to be completed in 2025.

### Others

#### CSF drainage

As SCPP is determined by the difference between MAP and intraspinal pressure, CSF drainage through a lumbar intrathecal catheter placed into the subarachnoid space is another method to maintain spinal cord blood flow. This procedure is commonly utilized for patients undergoing thoracoabdominal aortic aneurysm repair surgery, which potentially has a risk of spinal cord ischemia due to hypoperfusion from the important segmental artery connected to the anterior spinal artery [209]. A phase I clinical trial for acute SCI (NCT00135278) that commenced in 2006 indicated that CSF drainage did not result in any significant adverse events. However, it failed to demonstrate a significant difference in the ASIA motor score, likely due to the small sample size [210]. Following this trial, a phase II RCT was conducted from 2015 to 2019 (NCT02495545) to compare outcomes between MAP maintenance with CSF drainage and MAP maintenance alone. The results of this trial have not yet been published.

#### Therapeutic hypothermia

Therapeutic hypothermia is used in various medical scenarios to minimize secondary damage to the central nervous system. For instance, the 2020 American Heart Association Guidelines for cardiopulmonary resuscitation recommends that the target temperature for patients who achieve return of spontaneous circulation should be maintained between 32°C and 36°C for at least 24 h [211].

Therapeutic hypothermia is also regarded as a neuroprotective strategy for acute SCI. Several preclinical and clinical studies have suggested that this procedure might improve behavioral outcomes [212, 213]. A RCT comparing systemic hypothermia with standard treatment (NCT02991690) was initiated in 2017 and is expected to be completed by 2024. According to the interim report published in 2022 [214], preliminary data indicated that modest systemic hypothermia (33°C for 48 h) following acute SCI was not associated with an increased risk of complications. The results are anticipated to clarify the efficacy of therapeutic hypothermia.

## Examples of therapies at the advanced stages of translation

### Riluzole

Riluzole, a benzothiazole approved by the FDA for the treatment of amyotrophic lateral sclerosis, acts as a neuroprotective agent. It blocks sodium channels and reduces glutamate-associated excitotoxicity by decreasing glutamate release from the presynaptic terminal, preventing glutamate receptor hypofunction, and stimulating glutamate uptake [215]. Following SCI, voltage-sensitive sodium channels are constitutively activated, leading to increased intracellular sodium concentration, cellular swelling, and intracellular acidosis [216]. Additionally, the increase in intracellular sodium facilitates the influx of calcium ions through the Na^+^/Ca^2+^ exchanger, resulting in the extracellular release of excess glutamate and localized cell death. Riluzole is well-suited to inhibit these processes involved in secondary injury.

Based on promising results from several pre-clinical studies supporting the effectiveness of riluzole for acute SCI [217, 218], a phase I clinical trial for demonstrating the safety of riluzole in acute SCI (NCT00876889) was conducted between 2010 and 2012. This trial demonstrated that there were no serious adverse events associated with riluzole [219]. Additionally, patients with cervical SCI treated with riluzole had a significantly higher mean ASIA motor score at 90 days post-SCI compared to matched patients in the North American Clinical Trials Network SCI Registry (31.2 points vs. 15.7 points; *p* = 0.021). These encouraging results led to a double-blind phase IIb/III RCT, the Riluzole in Acute Spinal Cord Injury Study (RISCIS) (NCT01597518), initiated in 2014. Originally planned to enroll 351 patients, the trial was terminated in 2021 with 193 participants due to the COVID-19 pandemic. The primary efficacy outcome of the Upper Extremity Motor score at 180 days post-SCI showed no significant difference between the riluzole and control groups, likely due to insufficient power [157]. However, *post hoc* analysis revealed some hopeful results; for instance, the Upper Extremity and Total Motor score in the AIS C population treated with riluzole at 180 days post-SCI were significantly better than those without, according to multivariate linear regression models. Although this trial could not definitively determine the efficacy of riluzole, Fehlings et al. concluded that riluzole could be considered as one of therapeutic options in the clinical settings, given the lack of alternative pharmacological treatments for severe SCI. Currently improved techniques for trial design and handling the heterogeneity of patients will enhance future results.

Additionally, a phase III RCT for chronic cervical SCI (NCT01257828) was also conducted between 2012 and 2017. This trial did not show significant difference between the riluzole and control groups in the primary efficacy endpoint measured by the change in the modified Japanese Orthopaedic Association score at 6 months post-intervention [220]; however, the latest secondary analyses using a global statistical test showed a significant functional improvement at 1-year post-intervention in the riluzole group compared to the control group (in press). Riluzole remains a promising pharmaceutical treatment for SCI.

### Neuromodulation and stimulation

The main goal of rehabilitation strategies after SCI is to enhance functional recovery [221]. One possible way to achieve this goal is to strengthen the efficacy of the residual neuronal pathways [222, 223]. Electrical and magnetic neural stimulation induces significant and long-lasting neuroplastic effects that involve neuroplasticity markers [222, 223].

### Transcranial magnetic stimulation

Non-invasive repetitive transcranial magnetic stimulation (rTMS) has been applied to target sensory and motor function impairments, spasticity, and neuropathic pain [222]. The influence of rTMS in patients with SCI may confirm the hypothesis about the significance of the propriospinal system and other residual efferent pathways in the recovery of motor control [224]. Moreover, rTMS targeted at the motor cortex has suggested therapeutic potential in alleviating chronic neuropathic pain [225–228], indicating its beneficial effects in evaluating and enhancing motor function in SCI patients [224, 229] (Table 3).

**TABLE 3:** Summary of neurorehabilitation and stimulation strategies on spinal cord injury patients.

Approach	Treatment type	Study design	Stage of SCI	Sample size	Main outcome	References
Kinesiotherapy and rTMS in patients after Incomplete Cervical or Thoracic SCI	rTMS enhanced the corticospinal synaptic transmission	Clinical Research	Incomplete SCI at the C2–Th12 levels	26	Neurophysiological recordings produced significantly better MEP parameters in the K + rTMS group. This effect was sustained for at least 5 months	Wincek et al. [229]
5 Hz rTMS on sensory, motor, and autonomic function	Decreased motor cortical excitability	Clinical Research	Chronic SCI	15	Active motor threshold for the most caudally innervated hand muscle was increased with slightly improved hand function	Kuppuswamy et al. [224]
rTMS of the motor cortex on central pain after SCI	Depression scores were reduced	Clinical Research	Thoracic SCI	11	Long-term clinical effect on central pain. Pain scores were reduced and continued to improve at follow-up	Defrin et al. [226]
SCS on spasticity	Enhancing pre- and post-synaptic spinal inhibitory mechanisms	Clinical Research	Chronic SCI	12	Spasm was significantly reduced immediately after SCS, and spasticity measures were improved by 2 h post-induction	Hofstoetter, 2020 [230]
Non-invasive spinal cord electrical stimulation for arm and hand function in chronic tetraplegia	Improved the recovery of sensory function Decrease in the frequency and severity of muscle spasms Reduced pain	Clinical Research	Chronic Cervical SCI	65	Safe and effective for improving hand and arm function	Moritz et al. [231]
Exercise program on the rehabilitation of patients with SCI	Improve resistance and muscular strength	Clinical Research	Thoracic SCI	13	Positive impact on physical function	Durán et al. [232]
Targeted stimulation for restoration of motor and autonomic function in individuals with SCI	NA	Clinical Research	Thoracic SCI cervical SCI	47	Effective strategies for the concurrent recovery of the various effects associated with severe chronic SCI	Angeli et al. [233]
Targeted neurotechnology restores walking in SCI patients	Adaptive control of paralyzed muscles during overground walking, locomotor performance improved, regained voluntary control over paralyzed muscles and walk or cycle in ecological settings	Clinical Research	Chronic cervical SCI	3	Technological framework for improving neurological recovery and supporting the activities of daily living after SCI	Wagner et al. [234]
Recovery of overground walking after chronic motor complete SCI	Recovery of walking, standing, and trunk mobility	Clinical Research	Chronic SCI C4–T4	4	Intentional over-ground walking ability years after SCI	Angeli et al. [235]
Activity-dependent spinal cord neuromodulation rapidly restores trunk and leg motor functions after complete paralysis	Sufficient improvement to restore activities	Clinical Research	Chronic SCI	3	Activity-specific stimulation programs improved stand, walk, cycle, swim, and control trunk movements	Rowald et al. [236]
Walking naturally after SCI using a brain-spine interface	BSI enables natural control over the movements of legs to stand, walk and climb stairs	Clinical Research	Chronic SCI	1	Neurorehabilitation supported by the BSI improved neurological recovery	Lorach et al. [237]
Robot-assisted gait training improves walking function and activity in SCI	Improvements in gait distance, leg strength, and functional level of mobility	Clinical trials	Incomplete SCI	502	RAGT treatment is a promising technique to restore functional walking and improve locomotor ability	Nam et al. [238]
Robotic assisted gait training on ambulation and functional capacity in patients with SCI	Improvement in the walking index and functional independence measure scores	Clinical Research	Complete and Incomplete SCIs	88	Robotic-assisted gait training combined with conventional therapy is superior to conventional therapy in terms of gait function and level of disability	Yıldırım et al. [239]

Abbreviations: SCI, spinal cord injury; SCS, transcutaneous spinal cord stimulation; rTMS, repetitive transcranial magnetic stimulation; scES, spinal cord epidural stimulation; BSI, brain–spine interface; RAGT, robot-assisted gait training.

### Electrical stimulation

Electrical stimulation can accelerate axonal growth and myelination [240], stimulate neurons to discharge bioelectric signals to strengthen muscle contraction, and reconnect the neural network of the spinal cord [241]. Therefore, the baseline excitability of neural circuits is regulated by electrical stimulation, leading to action potentials within and between neural circuits by adjusting excitability to a precise level [242]. Electrical stimulation also enhances neurotransmitter release capacity via recruiting local neurotransmitters across synaptic sites by stimulating the amount of neurostimulation of afferent nerve fibers [242]. Moreover, epidural electrical stimulation leads to greater recovery of motor output after a severe SCI, and with intensive training and electrical stimulation, recovery of walking, standing, and trunk mobility can occur years after SCI [235]. Within a single day, activity-specific stimulation programs have enabled standing, walking, cycling, swimming, and control of trunk movements [236]. In addition, Transcutaneous Spinal Cord Stimulation (SCS) shows promise in reducing spasticity [230]. The application of this non-invasive spinal cord electrical stimulation technique is safe and effective for improving hand and arm function in individuals with cervical SCI (Table 3) [231].

Electrical stimulation and neuromodulation strategies show promise for enhancing motor function in SCI patients. Despite their potential, the use of electrical and magnetic stimulation in SCI rehabilitation faces several considerations and limitations, necessitating significant validation through clinical trials. Additionally, methodological variability across studies complicates the interpretation of outcomes. Standardization of stimulation parameters, patient selection criteria, and outcome measures are essential to ease meaningful comparisons and robust conclusions regarding stimulation effectiveness in SCI rehabilitation.

### Neurorehabilitation

Various types of motor training such as bicycling, swimming, and locomotor training decrease the inflammatory response, increase neurotrophins, and may strengthen spared functions and guide spinal reorganization [83]. Exercise has been shown to preserve muscle mass [243], restore motor and sensory function [232, 244, 245], induce synaptic plasticity [246], increase the concentration of neurotrophic factors in spinal and muscle tissue [247, 248], and reduce inflammation around the injured site [245] (Table 3).

Studies investigating the timing of exercise post-injury suggest it may yield advantageous or adverse consequences on the recovery outcomes [249–251]. Despite numerous studies, several questions remain unanswered regarding therapeutic tools, such as optimal rehabilitation timing, the most suitable intensity, duration, and frequency, as well as the best use of task-specific training for recovery of various functional modalities.

### Robot rehabilitation and brain–computer interfaces

In recent years, neural interfaces such as Brain-computer interfaces (BCIs) have used physiological brain activity to control external devices, thereby enabling severely disabled patients to interact with the outside environment [252]. Invasive and non-invasive BCI approaches have been used to promote neural control of a robotic arm [253] for patients with severe paralysis. This system has the potential to provide a new way of controlling their wheelchair [238, 239, 254].

All approaches to adaptive technology for patients with SCI have aimed to provide patients with control of their paralyzed limbs [233, 234, 237]. However, the possibility that similar systems could bring neuroplastic alterations that contribute to functional rehabilitation remains unclear. The technologies in combination with current pharmacological and neurostimulation approaches may offer a crucial pathway to motor recovery following SCI [255, 256].

## Ethical considerations

While the medical concerns of SCI such as neuropathic pain and spasticity have received significant attention, a smaller number of works have focused on ethical issues related to treatment and research in SCI, such as voluntary consent, patient welfare, transparency, medical decision-making, and the patient-physician relationship [257, 258]. The perspectives, priorities, and experiences of individuals living with SCI are influenced by social, environmental, clinical, and injury-associated aspects [259]. In addition, living with SCI is aggravated by a fair treatment concern and sociocultural factors at a systems level, such as a lack of accessible support, information, and rehabilitation as well as healthcare services, which create hindrance to reunification of research and clinical studies [260].

SCI research raises crucial ethical questions concerning participant welfare and the use of funding [257–260]. The approach of a consistent clinical trial plays a very important role in the reliability of the research, especially the inclusion and exclusion criteria, ethical issues, treatment uniformity, and informed consent [258, 259]. The inclusion and exclusion criteria to control the consistency of the trial must be developed based on the specific research content [259, 261]. Informed consent for clinical trials as well as the standardization of the operation procedures and rehabilitation treatment is necessary [261].

However, within SCI research, the concept of data sharing, meta-analysis, and the application of new statistical techniques such as recursive partitioning and the global statistical test show promise. A notable challenge is that publications focusing on preclinical research often present only a fraction of the generated data [262]. This limitation may be attributed, in part, to the constraints imposed by journals on word counts as well as the number of figures and tables allowed. Nonetheless, in recent years, various stakeholders within the SCI research community have actively advocated for publication standards and promoted the sharing of experimental data. Initiatives like The Open Data Commons for Spinal Cord Injury (ODC-SCI.org) have emerged, facilitating data sharing and enabling pooled data-driven discoveries while appropriately acknowledging the contributors of valuable SCI data [261].

Research involving individuals with SCI must prioritize patient well-being and minimize any potential harm or discomfort related to experimental procedures [263–265]. In addition, clear communication about the risk factors and potential benefits of research is essential; therefore, SCI patients should have access to information about the research process and its implications. Moreover, transparency regarding funding, prioritizing research subjects with the greatest potential to improve quality of life and develop new treatments, adhering to established standards of scientific integrity, accurately reporting findings, avoiding data manipulation, and community engagement to promote ethical conduct are key factors in clinical trial studies [263–265].

### The burden of SCI and inequality in the international context

The challenges faced by individuals with SCI across different countries and regions around the world include issues such as access to healthcare, rehabilitation services, and supporting devices. In many parts of the world, there is a lack of resources and support systems for people living with SCI, leading to increased vulnerability and inequality. Understanding and addressing these issues on a global scale is crucial for improving the quality of life and outcomes for individuals with SCI worldwide [266].

Unaddressed healthcare needs are significant for SCI patients, where people in low-income groups tend to be more affected. Among the barriers to meeting healthcare needs are healthcare cost, transportation, and service availability [266]. To improve the situation, a combination of measures from the health and social systems are required. Improving access to healthcare to ensure individuals with SCI have access to affordable and appropriate assistive technologies, specialized medical care, and rehabilitation services. This can improve functional outcomes and quality of life for all SCI patients regardless of their socioeconomic status. Providing equal access to education and employment opportunities for individuals with SCI and promoting public awareness to reduce stigma contributes to creating a more equitable and inclusive world for individuals living with SCI globally [266, 267].

## Future directions: combinatorial therapies

As each therapy seeks to target aspects of SCI, combinatorial approaches are now emerging to match the disease timeline and improve functional outcomes. Exemplifying the notion of combinatorial approaches to SCI can be done with the strategies currently available. Rehabilitative as well as FES (functional electrical stimulation) therapies rely on the presence of remaining neurons and parenchyma for the establishment of new synapses and subsequent functional recovery. Complementing rehabilitation and FES are early surgical intervention and stem cell therapies. Early surgical intervention prevents further secondary injury through reperfusion of the injured spinal cord, increasing the net preserved CNS tissue for synapse recruitment as well as reducing inflammation and scarring hostile to recovery. Similarly, stem cell therapy can be used for the replacement of lost cells in the CNS as well as creating a microenvironment conducive to repair through reduced inflammation. This potentially gives greater neuronal and synapse recruitment for better functional outcomes and impact from rehabilitation and FES.

Pharmaceutical options are currently limited but follow a similar trajectory, with each drug potentially playing a different role dependent on which aspect of SCI injury is targeted. The growing movement towards personalized medicine is highly applicable to the heterogeneous nature of SCI. There is a great degree of variability in effectiveness and perceived outcomes between treatments across patients. This has been highlighted particularly well by patient perceptions on the effectiveness of different treatments for chronic pain. Preferences have been shown for different opioid medications, diazepam, rehabilitative exercise, as well as massage – all to differing degrees [268, 269].

## Conclusion

There have been remarkable advancements in medical, surgical, and rehabilitative treatments for SCI. However, despite these advances, opportunities exist to develop reparative and regenerative approaches to enhance outcomes. Although it remains true that the outcomes in SCI improve in a very incremental fashion due to the complex nature of SCI pathophysiology, the advancements in techniques and strategies are impressively thorough and creative. By incorporating patient-centric approaches, insight into individual differences can guide current and emergent treatment as well as provide better autonomy to patients. Adapting each regenerative approach into a cohesive strategy in concert remains a difficult task yet even modest improvements in sensory and motor function returning to patients can be both meaningful and motivating in the face of a highly debilitating disease.
